# Polioencephalopathy in Eurasier dogs

**DOI:** 10.1111/jvim.16945

**Published:** 2023-12-02

**Authors:** Faye Rawson, Matthias Christen, Jeremy Rose, Emilie Paran, Tosso Leeb, Angela Fadda

**Affiliations:** ^1^ Langford Veterinary Services University of Bristol Bristol UK; ^2^ Institute of Genetics, Vetsuisse Faculty University of Bern Bern Switzerland; ^3^ Lumbry Park Veterinary Specialists Alton UK

**Keywords:** canine, movement disorder, neurodegenerative, polioencephalopathy

## Abstract

**Background:**

Polioencephalopathies secondary to inborn errors of metabolism have been described in dogs, but few genetically characterized.

**Objectives:**

Clinically and genetically characterize polioencephalopathy in a family of Eurasier dogs.

**Animals:**

Three Eurasier dogs (littermates) presented with early onset movement disorders (9 weeks in 2, 4‐6 months in 1). Progressive gait abnormalities were detected in 2 of the dogs, persistent divergent strabismus in 1, whereas consciousness and behavior remained intact in all dogs. One dog was euthanized at 25 months.

**Methods:**

Video footage was assessed in all dogs, and Dogs 1 and 2 had examinations and investigations performed. Whole genome sequencing of Dog 1 and further genetic analyses in the family were performed. A cohort of 115 Eurasier controls was genotyped for specific variants.

**Results:**

Episodes were characterized by generalized ataxia, as well as a hypermetric thoracic limb gait, dystonia, and irregular flexion and extension movements of the thoracic limbs. Magnetic resonance imaging of the brain in Dogs 1 and 2 identified symmetrical, bilateral T2 and fluid attenuated inversion recovery hyperintense, T1 hypo to isointense, nonenhancing lesions of the caudate nucleus, lateral and medial geniculate nuclei, thalamus, hippocampus, rostral colliculus and mild generalized brain atrophy. Genetic analyses identified a homozygous mitochondrial trans‐2‐enoyl‐CoA reductase (*MECR*) missense variant in all 3 dogs, and a homozygous autophagy‐related gene 4D (*ATG4D*) missense variant in Dogs 1 and 2.

**Conclusions and Clinical Importance:**

We describe a presumed hereditary and progressive polioencephalopathy in a family of Eurasier dogs. Further research is needed to establish the role of the MECR gene in dogs and the pathogenic effects of the detected variants.

**Abbreviations**
*ATG4D*
: autophagy‐related gene 4D
FLAIR: fluid attenuated inversion recovery
LA: lipoic acid
*MECR*
: mitochondrial trans‐2‐enoyl‐CoA reductase gene
MEPAN: mitochondrial enoyl CoA reductase protein associated neurodegeneration
MRI: magnetic resonance imaging
PE: polioencephalopathy

## 1 INTRODUCTION

Polioencephalopathy (PE) with multifocal bilateral and symmetrical gray matter lesions has been described both in people and rarely in animals and are typically manifestations of selected susceptibility of cellular subpopulations to metabolic aberrations such as nutritional deficiencies, toxicosis or inborn error of metabolism.^1^, ^2^, ^3^, ^4^, ^5^, ^6^, ^7^, ^8^, ^9^, ^10^, ^11^, ^12^, ^13^, ^14^ Among these latter conditions, mitochondrial disease is increasingly recognized in human medicine as a cause of PE resulting in varied clinical signs including cognitive regression, impairment of motor function and movement disorders.^15^, ^16^

Heterogeneity of clinical presentations represents a major challenge for identification of mitochondrial encephalopathies in people, and genetic testing is deemed crucial for early identification of disease.^17^ Likewise, genetic investigations have expanded understanding of uncommon encephalopathies in veterinary medicine, but to date very few causative genetic variants for mitochondrial encephalopathies have been identified in dogs.^14^, ^18^, ^19^, ^20^

Here we report the clinical findings and preliminary results of genetic analyses for 3 young and related Eurasier dogs affected by a PE with atypical manifestations, characterized by initial onset of movement disorder episodes and progressive impairment of motor function, but preserved consciousness and behavior. Our investigations support *MECR*:c.823A>G as a possible causative variant for this presumed hereditary and progressive PE in these littermates, which shares striking similarities with a rare form of childhood‐onset dystonia reported in individuals with *MECR* loss of function variants.^21^, ^22^, ^23^, ^24^ Prospective studies are warranted to investigate the association of *MECR* variants and neurodegeneration in dogs and to validate whether *MECR*:c.823A>G is causally involved in this previously unreported PE within the United Kingdom (UK) Eurasier population.

## 2 MATERIALS AND METHODS

### 2.1 Ethics statement

Institutional ethical approval was granted for this study from the University of Bristol Animal Welfare and Ethical Review Body (VIN‐22‐055). The collection of EDTA blood samples was approved by the Cantonal Committee for Animal Experiments (Canton of Bern; permit BE 71/19).

### 2.2 Animals

Blood samples were collected from 2 male intact Eurasier littermates (Dogs 1 and 2) presented to 2 separate UK‐based small animal referral centers with movement disorder episodes since 9 weeks of age, from a female intact littermate (Dog 3) described to have similar, but milder and exercise‐induced episodes at 4‐6 months, and from 3 additional, reportedly healthy (at the time of investigations), littermates and from both parents. An additional 115 Eurasier dog samples from the Vetsuisse Biobank were used as a control.

### 2.3 Genetic analyses

Deoxyribonucleic acid was extracted from EDTA blood samples using the Maxwell RSC Whole Blood DNA Kit on a Maxwell RSC instrument (Promega, Dübendorf, Switzerland). The DNA from the entire family consisting of both parents and 6 puppies was genotyped on illumina_HD canine BeadChips containing 220 852 markers (Neogen, Lincoln, NE, USA).

Homozygosity mapping was performed using PLINK v1.9, and parametric linkage analysis with an autosomal recessive inheritance model with full penetrance was performed using the Merlin software.^25^, ^26^ Both analyses were performed once under the assumption that only Dogs 1 and 2 were affected and once again for the hypothesis that Dogs 1‐3 all were affected.^25^, ^26^

A whole genome sequence of Dog 1 was generated with 21.8× coverage on a NovaSeq 6000 instrument after preparation of an Illumina TruSeq PCR‐free DNA library with an approximately 400 base pair insert size. Mapping and alignment to the UU_Cfam_GSD_1.0 reference genome assembly were performed as described,^27^ and the sequence data were deposited under the study accession PRJEB16012 and the sample accession SAMEA110415690 at the European Nucleotide Archive. For variant filtering, we used 955 control genomes of different dog breeds (Table S1).^27^

The *ATG4D*:c.1187C>T and the *MECR*:c.823A>G variants were genotyped in the 8 dogs from the family and 115 additional Eurasier dogs from the Vetsuisse Biobank by direct Sanger sequencing of PCR amplicons. The used primer sequences are presented in Table S2.

## 3 RESULTS

### 3.1 Clinical description

Two related male intact Eurasier puppies from the same litter were presented within a 1‐month period to 2 UK‐based referral centers for evaluation of recurrent neurological signs. A third female intact Eurasier puppy from the same litter was evaluated using videos provided of episodes that occurred at 4‐6 months of age.

**Dog 1:** A 21‐week‐old intact male puppy initially was presented with a 12‐week history of recurrent movement disorder episodes having been previously normal. The episodes appeared to coincide with overstimulation, excitement, and loud noises. Consciousness was retained throughout. The episodes initially were infrequent and characterized by generalized ataxia and a hypermetric thoracic limb gait, dystonia, irregular flexion and extension movements of the thoracic limbs, resembling episodes of ballism and choreoathetosis as occur in people. Furthermore, when trying to ambulate during episodes, Dog 1 would appear to advance entirely using its pelvic limbs because of increased extensor tone in the thoracic limbs which were consequently outstretched beyond the tip of the nose with normal stepping movement prohibited. Frequency and severity of the episodes increased over time to multiple times per day, with each episode lasting up to several hours and resulting in marked exercise limitation. General physical and neurological examinations were unremarkable initially, but soon after the start of the consultation Dog 1 appeared to progress into an episode as previously described. In addition, a marked divergent ventrolateral strabismus of the left eye and decreased peripheral vision was detected (Supplementary video 1).

**Dog 2:** A 15‐week‐old intact male puppy was presented with a 6‐week history of abnormal movement disorder episodes which appeared initially to be exercise induced with intermittent hopping on the thoracic limbs and collapsing on the pelvic limbs, and later with a head tilt, strabismus, and increased tone in the thoracic limbs. During episodes, Dog 2 appeared conscious, with each episode lasting up to 30 minutes. Since the initial episodes, episode frequency increased and resulted in moderate exercise limitation. General physical examination was unremarkable at the initial evaluation. On neurological examination, Dog 2 was mildly tetraparetic with a subtle pelvic limb ataxia (Supplementary video 2).

**Dog 3:** An intact female puppy was noted to have movement disorder episodes if exercised for more than 20 minutes, with video footage provided. Dog 3 was not examined and did not have investigations. Video footage showed episodes similar to those of Dogs 1 and 2, characterized initially by increased tone in all 4 limbs, more pronounced in the thoracic limbs, leading to abnormal posture, with extended neck and head resembling thoracic limb dystonia and an abnormal gait (similar to Dog 1). Also, Dog 3 would advance entirely by using the pelvic limbs because of increased extensor tone in the thoracic limbs, which consequently were outstretched beyond the tip of the nose with normal stepping movement prohibited. Because of the association with exercise duration, Dog 3 was managed with exercise limitation, which appeared to substantially decrease the frequency of the episodes (Supplementary video 3).

### 3.2 Clinical investigations in Dogs 1 and 2

Complete blood counts and serum biochemistry were unremarkable other than age‐related changes (low total protein concentration, increased ALP activity, serum total hypercalcemia and hyperphosphatemia in Dog 1 and increased ALP activity and hyperphosphatemia in Dog 2). Both had normal bile acid stimulation test results, and Dog 1 had a mildly increased ammonia concentration of 59.8 μmol/L (normal, 0‐50 μmol/L). Both had mildly increased CK activity and normal AST activity (Dog 1: CK, 552 U/L; Dog 2: CK, 315 U/L; reference interval, 75‐230 U/L). Toxoplasma and Neospora serology results were negative. Cobalamin, folate and thiamine serum concentrations were within normal limits. Venous blood gas analysis was unremarkable, with no evidence of hyperlactatemia at rest or after exercise. Urine organic acids analysis results in Dog 1, performed at an external human medical laboratory, were comparable to those of a clinically healthy control dog.

Electrodiagnostic studies performed in Dog 2 were normal. Electromyography of the left pelvic limb was normal, with normal left sciatic tibial motor conduction velocity (72 m/s), compound motor action potential of the left sciatic tibial nerve (proximally 14.1 mV) with normal wave form (stimulating at the sciatic notch and recording at the interosseous), distally 22.3 mV with normal wave form (stimulating proximal to the hock and recording at the interosseous) and F‐wave latency of the left sciatic tibial nerve (18.18 ms; reference interval, 18.3 ms). Repetitive nerve stimulation of the left common peroneal while recording at the cranial tibial was normal (3 Hz + 2.1% and 10 Hz + 2.0%).

Cisternal cerebrospinal fluid (CSF) analysis was normal. Toxoplasma and Neospora were not detected on PCR of the CSF in either dog.

**FIGURE 1:**
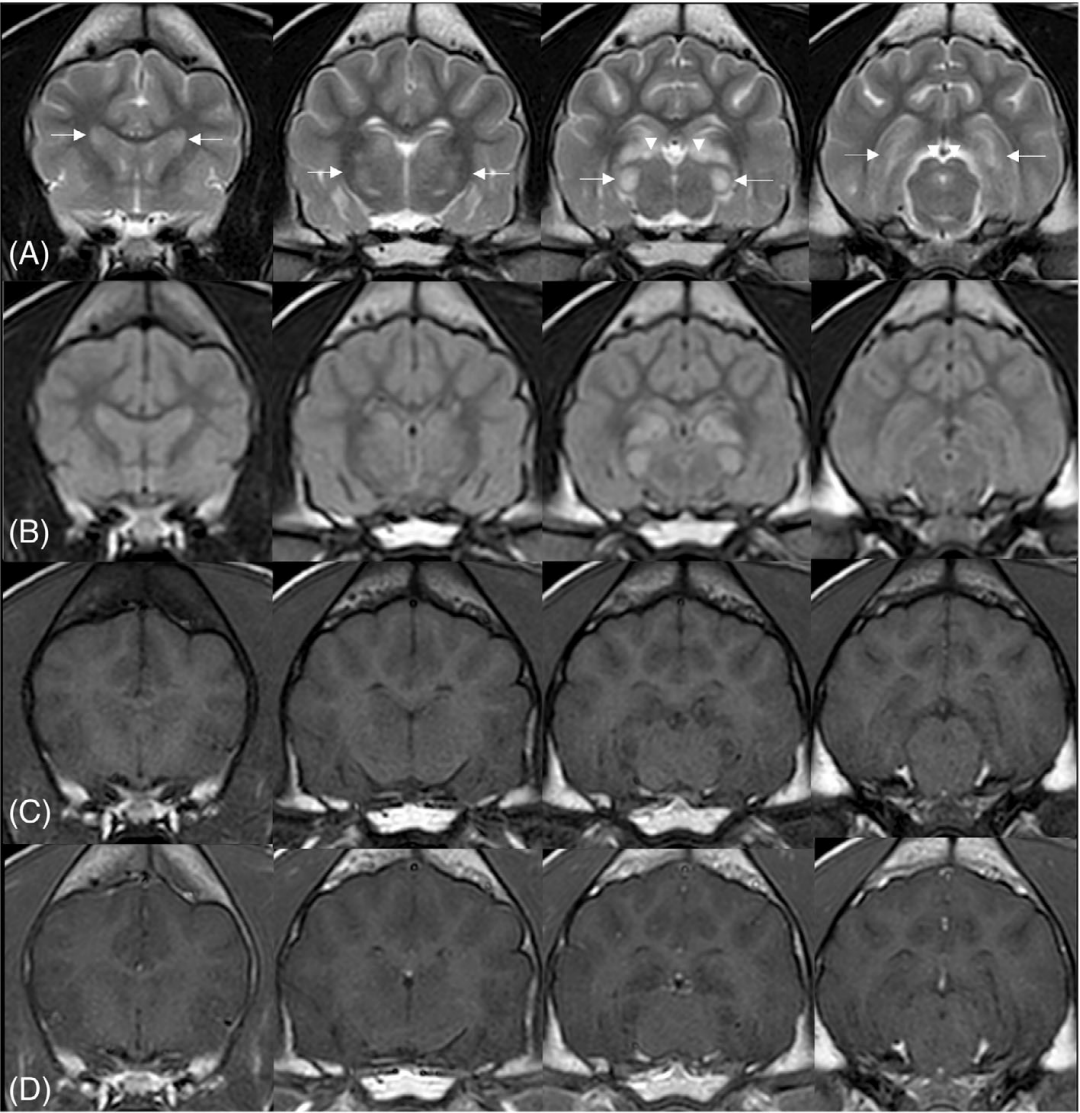
Selected MR images of the brain of Dog 1 in T2w (A), FLAIR (B), T1w (C), and T1w post contrast (D) sequences. The caudate nuclei are shown in the first column (arrows); the thalamus in the second column (arrows); the medial geniculate nuclei (arrows) and lateral geniculate nuclei (arrowheads) in the third column; the hippocampi (arrows) and rostral colliculi (arrowheads) in the fourth column. These areas are symmetrically bilaterally T2/FLAIR hyperintense, T1 hypo to isointense, and noncontrast enhanced. The thalamus and hippocampus are more affected in Dog 1 compared to Dog 2.

**FIGURE 2:**
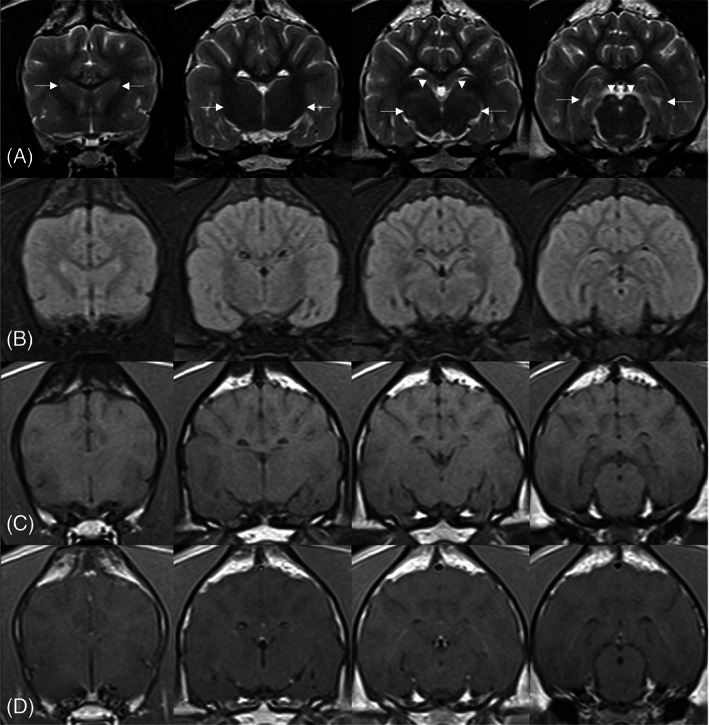
Selected MR images of the brain of Dog 2 in T2w (A), FLAIR (B), T1w (C), and T1w post contrast (D) sequences. The caudate nuclei are shown in the first column (arrows); the thalamus in the second column (arrows); the medial geniculate nuclei (arrows) and lateral geniculate nuclei (arrowheads) in the third column; the hippocampi (arrows) and rostral colliculi (arrowheads) in the fourth column. These areas are symmetrically bilaterally T2/FLAIR hyperintense, T1 hypo to isointense, and noncontrast enhanced. Dog 2 has milder abnormal signal at these locations; in particular the thalamus and hippocampus are less affected compared to Dog 1. The rostral colliculus is more affected in Dog 2 compared to Dog 1.

Dogs 1 and 2 had magnetic resonance imaging (MRI) at their respective referral centers. The images were acquired using 1.5T MRI scanners (Philips Ingenia CX 1.5T, Philips Medical Services, Eindhoven, Netherlands for Dog 1 and Siemens Magnetom Essenza, Siemens Healthcare GmbH, Erlangen, Germany for Dog 2). Dog 1 (Figure 1) had more severe lesions than Dog 2 (Figure 2), but both had similar symmetrical bilateral multifocal T2 and fluid attenuated inversion recovery (FLAIR) hyperintense, T1 hypo‐ to isointense, noncontrast enhancing lesions throughout the gray matter of the dorsolateral aspect of the caudate nucleus, lateral and medial geniculate nuclei, thalamus, hippocampus, and rostral colliculus. The thalamus and hippocampus were more affected in Dog 1. The rostral colliculus was more affected in Dog 2 (Figures 1 and 2). There was no evidence of hemorrhage or restricted diffusion in either of the dogs. The CSF signal around the forebrain, within the cerebral sulci, was more pronounced compared to published data.^28^

After investigations, treatment was started in Dogs 1 and 2. Clonazepam 0.2 mg/kg PO q8h in Dog 1 and 0.4 mg/kg PO q8h in Dog 2 resulted in a decrease in episode frequency. Additional attempts at increasing the dose were made because of incomplete resolution of the clinical signs, but Dog 1 exhibited marked ataxia and sedation. No response was seen with thiamine supplementation. Carnitine was administered to Dog 1, but during this treatment trial, Dog 1 deteriorated transiently with difficulty ambulating and treatment was quickly discontinued. Anti‐inflammatory doses of glucocorticoids (prednisolone 0.5 mg/kg PO a24h) were used in Dog 1 with no response. No response was seen when the dogs were treated with chlorphenamine. Both dogs were fed Purina PRO PLAN NC Neurocare. Dog 2 was treated with Aktivait (Vetplus) because of the lipoic acid it contains, but it was discontinued because of a lack of response.

### 3.3 Follow‐up

At the 1‐year follow‐up, Dog 1 showed evidence of generalized muscle atrophy (suspected secondary to glucocorticoid treatment), most notable over the temporal muscles and hindquarters. On neurological examination Dog 1 was quiet, but responsive, and borderline ambulatory with difficulty rising and walking, falling to either side after a few steps, with a hypermetric gait in all 4 limbs when able to ambulate, and a low head carriage. Paw placement was intact in all 4 limbs, with hypermetric hopping responses in the thoracic limbs. Spinal reflexes were intact. Cranial nerve examination identified spontaneous divergent ventrolateral strabismus bilaterally with wide excursions of the head, and intermittent menace response bilaterally, decreased when menacing from both lateral visual fields. Spinal palpation was unremarkable (Supplementary video 4).

At the time of writing, the dogs were 26 months old with mild progression of the disease in Dog 1, managed with restricted exercise and ongoing clonazepam treatment. Dog 2 was euthanized at 25 months of age at the referring veterinary practice because of a prolonged movement disorder episode resulting in suspected heat stroke and a persistent comatose mental status after treatment. Dog 3 appeared well controlled at 15 months of age with exercise restriction implemented when exercise was identified as a trigger (Supplementary video 5).

### 3.4 Genetic analyses

Because of the occurrence of a neurological phenotype in several puppies from the same litter with healthy parents, we hypothesized a monogenic autosomal recessive mode of inheritance. Because of the phenotype in Dog 3 appeared milder than in Dogs 1 and 2, we considered the possibility of additional modifier loci that could explain the differences in phenotype severity between Dogs 1 and 2 (severe) in contrast to Dog 3 (mild). We therefore performed the genetic analyses in 2 versions, the first scenario only classified Dogs 1 and 2 as affected, whereas the second scenario classified Dogs 1‐3 as affected. Combined linkage and homozygosity mapping resulted 10 intervals spanning 103 Mb when only Dogs 1 and 2 were classified as affected (Table S3). The same analyses resulted in 10 different intervals spanning 84 Mb when Dogs 1‐3 were classified as affected (Table S4).

The genome of Dog 1 was sequenced and searched for homozygous private variants in the aforementioned critical intervals by comparing the variants with 955 control genomes (Supplementary Tables S1 and S5).

The analysis identified a single private homozygous variant with likely functional impact on a protein in each of the critical intervals. An *ATG4D* variant, XM_038567309.1:c.1187C>T, was identified in the critical interval derived from Dogs 1 and 2, whereas a *MECR* variant, XM_038531348.1:c.823A>G, was found in the critical intervals resulting from the second analysis, in which Dogs 1‐3 were classified as affected. Both variants are predicted to lead to an exchange of a single amino acid in the encoded proteins, XP_038423237.1:p.(Ala396Val) and XP_038387276.1:p.(Met275Val).

**FIGURE 3:**
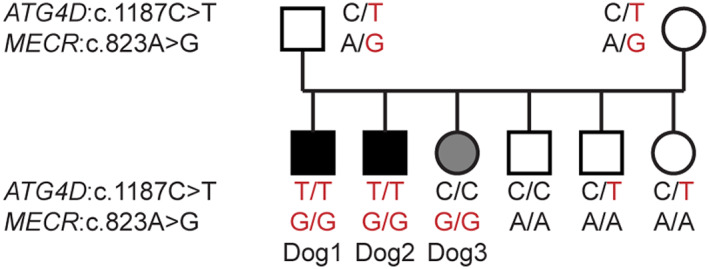
Pedigree of the Eurasier family showing the expected cosegregation of the genotypes at the 2 variants for the more severe phenotype in Dog 1 and 2, and the milder phenotype in Dog 3 assuming autosomal recessive inheritance.

Both variants were genotyped in the entire family and the genotypes cosegregated as expected with the severe (*ATG4D* and *MECR*) or mild neurological phenotype (*MECR* only; Figure 3). Both mutant alleles were not present in 115 additionally genotyped Eurasier dogs without reports of similar neurologic disease.

## 4 DISCUSSION

We identified a homozygous missense variant in *MECR* as a possible candidate causative variant for a previously unreported presumed hereditary and progressive PE in 3 littermate Eurasier dogs. An additional homozygous missense variant in *ATG4D* was identified in the 2 most severely affected dogs, possibly suggesting additive effects.

The *MECR* gene encodes mitochondrial trans‐2‐enoyl‐coenzymeA‐reductase, a protein involved with the synthesis of lipoic acid (LA) in eukaryotic cells. Lipoic acid is essential for the activity of mitochondrial fatty acid synthesis.^29^ Inborn error of metabolism affecting LA synthesis resembles typical mitochondrial disorders by the involvement of organs with high energy demands and susceptibility to oxidative stress such as the basal ganglia and optic nerves. Specific disruption of the *MECR* gene caused neurodegenerative changes and Purkinje cell loss in a recently established mouse model, further supporting the crucial role of this gene and mitochondrial fatty acid synthesis for cellular metabolism and survival in multiple neuronal systems.^30^

Although no information on the functional impacts of the *MECR* gene exists in dogs to date, in our 3 dogs, clinical signs and radiological findings share striking similarities with those reported in people with *MECR* variants and childhood‐onset dystonia and optic atrophy.^21^, ^22^ Children affected by this rare, progressive and debilitating neurodegenerative disease, show early occurrence of dystonia, decreases in balance and motor function, with or without vision loss and difficulties in communication (dysarthria) as the disease progresses. Magnetic resonance imaging identifies bilateral hyperintense T2W signal in the caudate and putamen nuclei in these patients, and although clinical and MRI findings overlap with those of other mitochondrial encephalopathies, it differs by the relative sparing of cognition and absence of additional organ involvement and typical mitochondrial biomarkers.^22^

Similarly, clinical presentation in our 3 dogs is atypical, reflecting simultaneous involvement of multiple neuronal systems, with movement disorder (dystonia/ballism) and progression of cerebellar signs, but no cognitive decline or behavioral changes. Moreover, these dogs lack systemic signs or laboratory changes suggestive of multiorgan failure.

Contrary to what is reported in individuals affected by childhood‐onset dystonia and optic atrophy, we have not identified optic atrophy in any of the dogs so far. Although this may be a result of the early stage of disease in these dogs, lack of optic nerve involvement is not entirely atypical for individuals with *MECR* variants, and because of the heterogeneity of clinical phenotypes occurring in children, research recently has suggested replacing the term childhood‐onset dystonia and optic atrophy with mitochondrial enoyl CoA reductase protein‐associated neurodegeneration (MEPAN).^21^, ^22^

Currently there is no specific and curative treatment for encephalopathies not associated with a nutritional deficiency.^31^ However, dietary supplementation with LA or its precursor octanoic acid is recommended in people with *MECR* variants to halt or slow disease progression. Although only attempted in 1 dog, LA supplementation did not appear to modify the course of the disease in this Eurasier, which also may suggest that differences in biochemical pathway disruption with mutated *MECR* gene exist between the 2 species. Additional treatment trials were performed in both Dogs 1 and 2 in attempts to address 2 of the 3 major neurotransmitter systems identified as targets for medical treatment in humans with dystonia: GABAergic (clonazepam) and cholinergic (chlorphenamine, an H_1_ antihistamine agent with anticholinergic properties).^32^ Benzodiazepines also have been used previously in both dogs with a paroxysmal hypertonicity disorder and in humans with akathisia.^33^, ^34^ Unfortunately, no response was seen to chlorphenamine treatment in Dogs 1 and 2, which could have warranted treatment using a more potent anticholinergic agent such as diphenhydramine.^35^ The promising response to benzodiazepines could warrant further treatment trials with antiepileptic medications. Likewise, dopaminergic treatment such as L‐dopa was not used in these dogs.^36^, ^37^

A further homozygous missense variant in *ATG4D* was identified in Dogs 1 and 2. The *ATG4D* gene encodes a poorly characterized cysteine protease belonging to the macroautophagy pathway. The *ATG4D* variations result in disruption of neuronal homeostasis, particularly in the axons and Purkinje cells, structures known to be dependent on basal autophagy.^38^, ^39^, ^40^, ^41^ An *ATG4D*:p.Ala430Thr variant previously has been associated with a recessive vacuolar storage disease in the Lagotto Romagnolo.^40^ Although in these 3 Eurasier dogs clinical signs also support abnormal function of the cerebellum, some clear disparities exist both clinically and in MRI studies between the Lagotto Romagnolo and our dogs with a variant in the *ATG4D* gene. This difference might be attributable to different effects of the p.Ala430Thr variant in Lagotto Romagnolo and the p.Ala375al variant in Eurasier dogs. Furthermore, additive effects of the *MECR* and *ATG4D* missense mutations cannot be excluded in Dogs 1 and 2, which experienced a severe clinical course.

Finally, clinical presentation and imaging characteristics of the dogs described here were not consistent with the previously described cerebellar hypoplasia syndrome in the Eurasier breed caused by deficiency in the very low density lipoprotein receptor (VLDLR:c.1713delC).^42^

Our study had some limitations, including the lack of clinical evaluation and investigations in Dog 3 (reliance on video footage and owner history) and lack of histopathologic diagnosis in any of the cases. Similarly, skin biopsies were not performed to assess dermal apocrine gland changes as reported in the Lagotto Romagnolo with *ATG4D* mutations, or for establishment of fibroblast cell lines culture to allow mitochondrial activity evaluation in affected dogs.^22^, ^40^ Additional work would include functional testing of these variants to confirm their impact in an in vivo system, such as yeast complementation assays, as previously reported for the *MECR* variant in humans.^22^ Finally, our genetic analyses included 115 control Eurasier dogs that were not from a UK‐based population. Including dogs from the UK possibly would have helped investigate transmission risks of *MECR* associated PE, largely owing to the small breeding population in the UK. Doing so was not possible, because of the very low number of samples from Eurasier dogs available in UK Biobanks.

To our knowledge, our is the first description of PE with early onset of movement disorders associated with a *MECR* variant in dogs. Because clinical manifestations occurred in multiple littermates and closely resemble those occurring in children with *MECR* variants, a genetic component is strongly suspected, with *MECR*:c.823A>G a potential candidate disease‐causing variant. More marked clinical signs are present when dogs simultaneously carried mutant alleles at *ATG4D*:c.1187C>T in a homozygous state, possibly suggesting additive effects.

Our results support further research elucidating the potential functional impact of *MECR* variants and their links to neurodegenerative disorders in dogs. Likewise, in view of a large amount of inbreeding in the small population of UK Eurasiers, additional studies to investigate the frequency and distribution of PE‐associated variants within this population are warranted to promote responsible breeding strategies.

## CONFLICT OF INTEREST DECLARATION

Authors declare no conflict of interest.

## OFF‐LABEL ANTIMICROBIAL DECLARATION

Authors declare no off‐label use of antimicrobials.

## INSTITUTIONAL ANIMAL CARE AND USE COMMITTEE (IACUC) OR OTHER APPROVAL DECLARATION

Institutional ethical approval was granted for this study from the University of Bristol Animal Welfare and Ethical Review Body (VIN‐22‐055). The collection of EDTA blood samples was approved by the Cantonal Committee for Animal Experiments (Canton of Bern; permit BE 71/19).

## HUMAN ETHICS APPROVAL DECLARATION

Authors declare human ethics approval was not needed for this study.

## Supporting information


**Figure S1.** Genotypes.

**Figure S2.** Genotypes.

**Video 1.** Dog 1 at 4.5‐months.

**Video 2.** Dog 2 at 4‐months.

**Video 3.** Dog 3 at 5‐months.

**Video 4.** Dog 1 at 12‐months.

**Video 5.** Dog 3 at 15‐months.

**Table S1.** Accession numbers of 956 dog genome sequences. The affected Eurasier is highlighted in yellow. The other 955 genome sequences were used as controls in variant filtering.

**Table S2.** Primer sequences used for amplifying positions around candidate variants in the canine ATG4D and MECR genes. Coordinates refer to the UU_Cfam_GSD_1.0 reference genome assembly.

**Table S3.** Linkage‐Homozygosity_2cases.

**Table S4.** Linkage‐Homozygosity_3cases.

**Table S5.** Summary of number of candidate variants remaining for analysis after several filtering steps. Linkage and homozygosity mapping were performed once for the hypothesis, that 2 dogs are affected, and once for 3 affected dogs. A variant was considered a functional candidate, if search in available databases (eg, pubmed, OMIM, Mouse genome informatic database) showed association of an affected gene with similar neurological phenotypes.
